# Determinants of infant mortality for children of women prisoners: a longitudinal linked data study

**DOI:** 10.1186/s12884-018-1840-z

**Published:** 2018-06-01

**Authors:** Caitlin McMillen Dowell, Gloria C. Mejia, David B. Preen, Leonie Segal

**Affiliations:** 1 Health Economics and Social Policy Group, Centre for Population Health Research, Sansom Institute, School of Health Sciences, University of South Australia, GPO Box 2471, Adelaide, SA 5001 Australia; 2 Centre for Health Services Research, School of Population and Global Health, University of Western Australia, Crawley, WA Australia

**Keywords:** Infant mortality, Women prisoners, Linked data, Australia

## Abstract

**Background:**

There is limited information on the determinants of infant mortality outcomes for the children of women prisoners. This study aimed to explore determinants of infant mortality for Indigenous and non-Indigenous children, with a specific focus on maternal imprisonment during pregnancy as a risk factor.

**Methods:**

Using linked administrative data we obtained a longitudinal sample of 42,674 infants born in Western Australia between October 1985 and June 2013. Data were analysed by maternal contact with corrective services, including; (i) imprisonment during pregnancy, (ii) imprisonment before (but not during) pregnancy, (iii) imprisonment after birth, (iv) community-based correctional orders (but no imprisonment), and (v) no corrections record. Infant mortality rates were calculated. Univariate and multivariate log-binomial regression was undertaken to identify key demographic and pregnancy-related risk factors for infant mortality. Risk factor prevalence was calculated for infants by maternal corrections history.

**Results:**

430 Indigenous and 116 non-Indigenous infants died aged 0–12 months. For singletons, infant mortality rates were highest in Indigenous infants with mothers imprisoned during pregnancy (32.1 per 1000) and non-Indigenous infants whose mothers were first imprisoned after birth (14.2 per 1000). For all Indigenous children, the strongest determinants of infant mortality were: abruptio placentae and other placental disorders (RR = 2.85; 95%CI 1.46–5.59; *p* = 0.002), maternal imprisonment during pregnancy (RR = 2.55; 95%CI 1.69–3.86; *p* < 0.001), and multiple gestation (RR = 2.29; 95% CI1.51–3.46; *p* < 0.001). Indigenous and non-Indigenous infants with mothers imprisoned at any time, and particularly before or during pregnancy, experienced higher prevalence of key pregnancy risk factors.

**Conclusions:**

This is the first comprehensive study of the determinants of infant mortality for children of women prisoners. Infants with any maternal corrections history, including community-based orders or imprisonment outside of pregnancy, had increased infant mortality. Indigenous infants whose mothers were imprisoned during pregnancy were at particular risk. There was a low incidence of infant death in the non-Indigenous sample which limited the investigation of the impact of the specific aspects of maternal corrections history on infant mortality. Non-Indigenous Infants whose mothers were imprisoned before or during pregnancy experienced higher prevalence of pregnancy risk factors than infants of mothers first imprisoned after birth.

**Electronic supplementary material:**

The online version of this article (10.1186/s12884-018-1840-z) contains supplementary material, which is available to authorized users.

## Background

Women prisoners constitute a highly vulnerable population which is exposed to multiple and complex risk factors, including domestic violence, substance abuse, poverty, discrimination and mental illness, placing them and their children at risk of poor pregnancy and health outcomes [1]. However, few reports, exist which have investigated pregnancy outcomes for children of women prisoners [2, 3]. Of those studies that have investigated pregnancy outcomes for infants, the main outcomes investigated include preterm delivery and low birth weight [3]. The few studies that report on infant death have been limited by small numbers of events, in part due to the size of the cohort sampled [3, 4].

While the international literature on the pregnancy outcomes of prisoners is equivocal, a review of studies from across the United States (US), United Kingdom and Europe found women imprisoned in pregnancy generally had poorer maternal and infant outcomes than community controls, and better outcomes than disadvantaged community controls [3]. It has been concluded that this may indicate that imprisonment in pregnancy may be beneficial for some pregnancy outcomes [3]. The only Australian study on pregnancy outcomes of women prisoners found, however, that women imprisoned during pregnancy did not have better perinatal outcomes than women imprisoned at times other than pregnancy [4]. Thus the unique context of the justice systems and prisoner populations in specific jurisdictions limit the transferability of findings across jurisdictions in the absence of an understanding of the determinants of pregnancy outcomes for women prisoners.

Studies in the US have found racial differences in the pregnancy outcomes of women prisoners [5]. In Australia, research on the pregnancy outcomes of Indigenous women prisoners is lacking despite their overrepresentation in the prison population. In Western Australia, for example, Indigenous peoples represent 4% of the general population but 46% of the female prison population [6, 7]. This reflects the high levels of social and economic disadvantage and discrimination experienced by Indigenous peoples in Australia [8]. Similarly, Indigenous peoples in Australia experience poorer pregnancy outcomes compared with non-Indigenous mothers, and while infant mortality rates have been improving for both Indigenous and non-Indigenous populations across time, the racial disparity remains [9, 10].

Across 1980 to 2001, infant mortality rates in Western Australia declined for both Indigenous (25.0 in 1980–84 to 16.1 in 1998–2001) and non-Indigenous infants (8.4 in 1980–84 to 3.7 in 1998–2001) [10]. Some important changes that occurred across the past 30-years include improved transport for rural and remote pregnant women, immunisation of infants, prevention of Sudden Infant Death Syndrome [10], as well as increased parental employment and maternal education [11]. Although the overall rates of infant mortality declined between 1980 and 2001, the disparity between Indigenous and non-Indigenous populations increased from a Relative Risk of 3.0 (95%CI 2.5–3.6) in 1980–84 to 4.4 (95%CI 3.5–5.5) in 1998–2001 [10]. For Indigenous infants, postneonatal mortality was higher than neonatal mortality, a pattern that indicates the impact of socioeconomic disadvantage and marginalisation on infant mortality outcomes [10].

The primary objective of this study was to explore determinants of infant mortality for Indigenous and non-Indigenous children, with a focus on whether maternal imprisonment during pregnancy is a risk factor for infant mortality. The specific aims of the study were to determine: infant mortality rates for Indigenous and non-Indigenous infants of mothers with different corrections histories; the key demographic and pregnancy-related risk factors which may contribute to infant mortality in Indigenous and non-Indigenous populations; the importance of maternal corrections history as a determinant of infant mortality after accounting for significant demographic and pregnancy-related risk factors; and the prevalence of key risk factors for infant mortality between infants with different maternal corrections histories.

## Methods

### Study design

We have used data from a large data linkage project to explore infant mortality within Indigenous and non-Indigenous children of mothers who have been exposed to the corrections system at different times in relation to their pregnancy. We compared infant mortality outcomes for children whose mothers had; (i) any period of imprisonment during pregnancy, (ii) imprisonment before (but not during) pregnancy, (iii) their first period of imprisonment after birth, (iv) community-based correctional orders (but no imprisonment), or (v) no corrections record at any time over the study period.

### Conceptual framework

Mosley and Chen’s [12] analytical framework for the study of child survival was adapted for the present study. The basis of this framework is that broader determinants necessarily act through biological pathways, or mechanisms, which impact before, during and after pregnancy on the healthy development of the fetus and infant, and ultimately on infant mortality. There is evidence that adverse events experienced before and during pregnancy can impact on fetal development and result in increased risk of poor infant and childhood health outcomes [13–17].

Our adaptation of this framework first groups together key demographic factors, including birth year, sex, Indigenous status, socioeconomic status, and geographical remoteness. The second grouping includes baseline pregnancy risk factors, such as multiple gestation, birth spacing < 18 months, maternal age, and parity, which are largely unmodifiable from the commencement of pregnancy [18]. The third grouping includes key pregnancy complications that might be a precursor of infant mortality, such as nutritional deficiencies, placental disorders, prematurity, and infection [19]. The last group includes other maternal factors and exposures which are known risk factors for infant mortality or may indicate maternal vulnerability or household dysfunction, such as substance use or mental health related service contacts, external causes of injury, and having other children in contact with the child protection system.

### Data sources

Data were obtained through the Western Australian Data Linkage System (WADLS). The WADLS uses highly-accurate computerised, probabilistic matching with clerical review to create linkages within and between administrative data collections across a range of Western Australian government agencies [20]. The Western Australian Data Linkage Branch conducted the linkage and provided de-identified data extracts. Records were extracted from the Midwives Notifications System, Birth Registrations, Death Registrations, Department of Justice, Hospital Morbidity Data System Collection (HMDC), Mental Health Information System (MHIS), and Department of Communities: Child Protection and Family Support (CPFS) data collections. These are all statutory State-wide data collections with complete coverage.

The Birth Registration and Midwives Notifications System data provided social and demographic characteristics of mothers and children at time of birth. Mortality data include all deaths registered in Western Australia. The Department of Justice data collection includes all custodial records for offenders held in Western Australian prisons and records for offenders on community-based correctional orders. Data excluded unsentenced individuals detained in police stations and courts, immigration detention centres, and mental health facilities. The HMDC includes all inpatient records for Western Australian public and private hospitals and day surgeries. The MHIS includes presentations to all inpatient and public community mental health services. The CPFS data include all reports of concerns for child welfare made to the child protection system and the details of investigations, protection applications and orders as well as placements in out-of-home care.

The Death Registrations, HMDC, and MHIS use the International Classification of Diseases (ICD), to classify cause of death, diagnosis, or reason for health service contact, respectively. For HMDC records, we obtained one code for the principal diagnosis of the episode of care, and up to four codes for external causes of episodes of care. Over the study period, the ICD 9th Revision with Clinical Modification (ICD-9-CM) [21] related to services contacts before July 1999, and service contacts from that date used ICD 10th Revision with Australian Modification (ICD-10-AM) [22].

### Study population

The study population was drawn from a retrospective longitudinal cohort study of all liveborn children born in Western Australia from 1985 to 2011 whose biological mother was imprisoned at least once within 18-years after their birth. The cohort study population included a comparison group of children whose mother had no record of imprisonment from their date of birth to their 18th birthday, which was identified through the same data sources as the cohort and matched 3:1 to cohort children on Indigenous status, age and sex. Data on second-generation children, born between 1998 and 2014 to the female members of the cohort and comparison group, were also obtained.

**Fig. 1:**
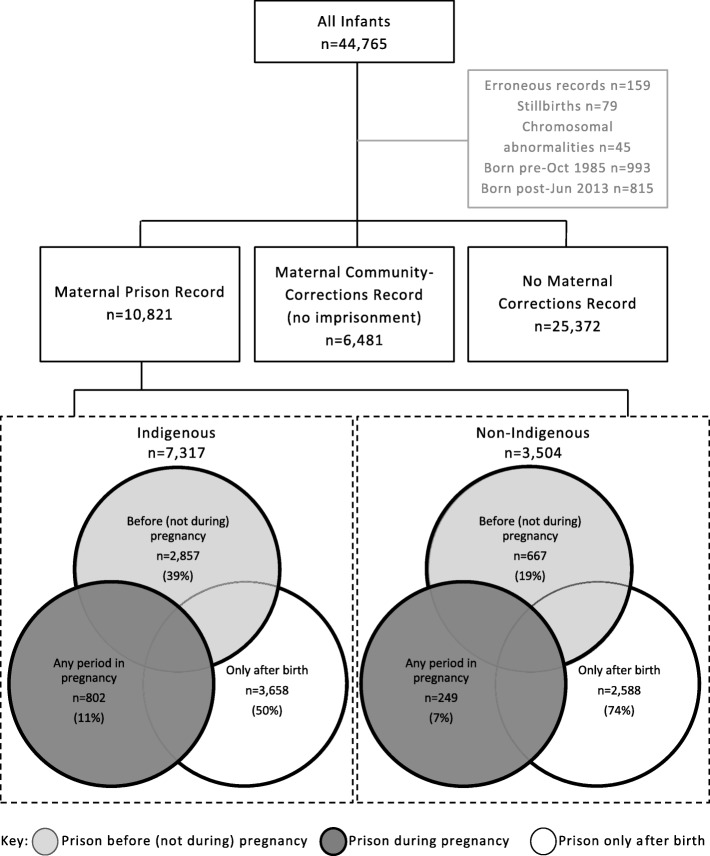
Selection of the study population and classification by maternal corrections history

Stillbirths (second-generation only) and infants with chromosomal abnormalities, identified through HMDC and death records, were excluded (Fig. 1). Erroneous records with multiple mothers or missing key information such as birthdate were removed. The final study population was restricted to children born from October 1985 to June 2013 (inclusive) to ensure pregnancy exposure and death data was available for all infants.

In total, there were 42,674 infants in the final study population, 37,469 from the first-generation (original cohort and comparison group) and 5205 from the second-generation (children of the original cohort and comparison group). Data from the birth and death registrations, midwives notifications records, and CPFS data were available for first- and second-generation children. Only first-generation children had HMDC record data. Mothers had corrections, HMDC, and MHIS data available.

### Definition of maternal corrections history

The study population was categorised into: a) infants whose mothers had a record of imprisonment at any time (*n* = 7317 Indigenous; *n* = 3504 non-Indigenous); b) infants whose mothers had community-based correctional orders but no record of imprisonment (*n* = 5828 Indigenous; *n* = 653 non-Indigenous); and c) infants whose mothers had no record within any Department of Justice database (*n* = 12,817 Indigenous; *n* = 12,555 non-Indigenous).

Imprisonment records covered prison stays of any length of time, and included unsentenced remandees detained before trial as well as sentenced prisoners. Infants of mothers who had a prison record at any time over the study period were further categorised based on the timing of their mother’s imprisonment in relation to their pregnancy. The first group included infants whose mothers had any record of imprisonment during pregnancy. The second group included infants whose mothers had imprisonment records in the period before, but not during, pregnancy. The third group included infants whose mothers first record of imprisonment only occurred after the child’s birth. These groupings are shown in Fig. 1. Classifications were based on mother’s prison reception dates and the infant’s birth date.

Community-based sentences may involve treatment or vocational programs, community service, and place restrictions on offenders. Breach of conditions while on community orders may result in imprisonment. Accordingly, women with community-based correctional orders are sentenced offenders, but may differ to women given custodial prison sentences in terms of severity or frequency of their offending and other individual factors. They are not exposed to the prison environment which generally places more stringent conditions on offenders and has different implications for them and their families.

The proportion of infants in the various maternal corrections history sub-groups (Fig. 1) relate only to the study sample and do not reflect the prevalence of these groups across the whole Western Australian population.

### Pregnancy and birth dates

Child month and year of birth was provided by the Midwives Notification System, or if missing from the Birth Registration data. As gestational age was not available, pregnancy start date was calculated as being nine months before the first day of the child’s birth-month.

### Definition of infant mortality

Infant mortality was defined as the death of a live born child under one year of age [23]. Full date of death was provided in the death registration data, however, birth data were available only for month and year of birth. Accordingly, infant mortality was defined as death within 12-full months after birth. For example, for a child born in January 2000, death on or before 31 January 2001 would be determined within the category of infant mortality.

### Demographic characteristics

The Birth Registration and Midwives Notifications System data provides social and demographic characteristics of mothers and children at time of birth, including sex, socioeconomic status and geographical remoteness. Sex was taken primarily from Midwives Data, or if missing from Birth Data.

Area-based socio-economic status of infant’s place of residence at time of birth was assigned using the Socio-economic Indexes for Areas (SEIFA) Index of Relative Socio-economic Disadvantage [24]. The smallest area of SEIFA reporting is Collectors District (CD) level, which is approximately 250 households or less in rural areas. Missing CD scores were imputed with mean CD-score by postcode before using broader area scores of SEFIA available for Statistical Local Area or Local Government Area [25].

The Accessibility/Remoteness Index of Australia (ARIA) [26] was used to classify the geographical remoteness of infant’s place of residence at time of birth. ARIA is derived from the measure of place of residence to populated locations and key services and classified as major cities, inner regional, outer regional, remote, and very remote locations. For the current study major cities and inner regional areas were combined given both have greater accessibility of relevant services [26].

Indigenous status for infants and mothers in the study populations was ascertained from the Derived Indigenous Status Flag variable generated by the WADLS using best-practice algorithms, which assess individuals’ Indigenous status across multiple data collections to enhance accuracy [27].

### Baseline pregnancy risk factors

Maternal birth date was determined using all available data sources. Maternal age was calculated as the age of mother at time of birth. Birth date was available for all siblings which enabled parity and duration of birth spacing to be determined. Multiple gestation pregnancies were also derived based on siblings having shared birth dates or through maternal or child HDMC data. Child-level HDMC data were not obtained for the second-generation children, however, as stillbirths were captured for the second-generation this assisted in identifying multiple gestation pregnancies.

### Pregnancy complications

The separate and combined effects of key pregnancy complications identified from maternal hospital records were evaluated. Pregnancy complications included the effects of infection-related hospitalisations, anaemia, diabetes, hypertension, preeclampsia, eclampsia, abruptio placentae, placenta previa, other placental disorders, premature rupture of membranes and renal disorders during pregnancy on infant mortality (Additional file 1) [19]. Complications were excluded due to low incidence or non-significance (*p* > 0.05), including hospitalisations for anaemia, diabetes, hypertension, preeclampsia, and eclampsia (Additional file 1).

### Other maternal risk factors and exposures

Maternal substance use (including alcohol) and poisoning-related service contacts during pregnancy were identified from HMDC and MHIS data. Maternal hospital admissions for any injuries from external causes, excluding substance use, self-harm and poisoning-related contacts were identified during pregnancy. Maternal hospital admissions for mental and behavioural disorders, self-harm, and mental health service presentations, both excluding substance use and poisoning-related contacts, were identified during pregnancy from HMDC and MHIS data. Having an older sibling in contact with child protection services during the infant’s pregnancy was also identified using sibling’s child protection data.

### Statistical analyses

All analyses were conducted using Stata Version 14.0. All analyses were stratified by Indigenous status of the infants.

Infant mortality rates (per 1000 population) were calculated for singleton infants by maternal corrections history. Prevalence of demographic and pregnancy-related risk factors were calculated for Indigenous and non-Indigenous populations. Log-binomial regression was used to calculate the Relative Risk of infant death for all univariate and multivariate analyses.

The strength of correlation between all variables of interest was assessed using Chi-square tests with Cramer’s V statistic. For variable pairs with a medium effect size (> 0.3) [28], one variable was excluded from further multivariate analysis on the basis of the univariate Relative Risk and level of statistical significance of each variable with infant mortality. Multivariate regression was then conducted for each grouping of variables (demographic factors, baseline pregnancy risk factors, pregnancy complications, and other maternal factors and exposures) separately with infant mortality, and variables that were not statistically significant (*p* < 0.05) were excluded from further analysis (Additional file 2). All remaining variables were entered with maternal corrections history into a full regression model (Model 1). Variables were removed by key groups; other maternal factors and exposures (Model 2), pregnancy complications (Model 3), baseline pregnancy risk factors (Model 4), leaving the combined effects of maternal corrections history and demographic factors with infant mortality. Goodness of model fit was assessed from the Akaike Information Criterion (AIC) value. There were insufficient numbers of non-Indigenous infant deaths whose mothers were imprisoned before or during pregnancy to undertake multivariate regression for non-Indigenous children.

Prevalence of key demographic and pregnancy-related risk factors, as determined from the univariate and multivariate analyses, were calculated for each maternal corrections history grouping and by Indigenous status.

## Results

In total, 546 infants in the sample died aged 0 to 12 months, between October 1985 and June 2014. There was a 2.39-fold risk of infant mortality for Indigenous infants compared to non-Indigenous infants (95% CI: 1.95–2.93, *p* < 0.001).

### Infant mortality by maternal corrections history

For singleton births, Indigenous infants whose mothers were imprisoned at any time had a significantly higher risk of death than infants of mothers with community corrections orders alone, or with no corrections history (Table 1). Indigenous infants whose mothers were imprisoned during pregnancy had the highest rates of infant mortality (32.1 per 1000), compared to infants whose mothers were imprisoned either only before pregnancy (22.1 per 1000) or after birth (23.6 per 1000) (Table 1).

**Table 1:** Infant mortality for singleton births, by maternal corrections history and Indigenous status

	Indigenous infants						Non-Indigenous infants					
	Survived	Died					Survived	Died				
	*n*	*n*	IMR^a^	RR	(95% CI)	*p*-value	*n*	*n*	IMR^a^	RR	(95% CI)	*p*-value
Maternal corrections history												
Prison (before pregnancy^b^)	2742	62	22.1	1.95	(1.45–2.62)	<.001	634	6	9.4	2.25	(0.97–5.22)	0.059
Prison (during pregnancy)	753	25	32.1	2.84	(1.87–4.31)	<.001	243	< 5	8.2	1.96	(0.48–8.00)	0.349
Prison (after birth only)	3481	84	23.6	2.08	(1.59–2.72)	<.001	2491	36	14.2	3.42	(2.24–5.23)	<.001
Community corrections	5588	94	16.5	1.46	(1.13–1.89)	0.004	628	8	12.6	3.02	(1.44–6.33)	0.003
No corrections*	12,400	142	11.3				12,186	51	4.2			

Note Excludes 591 Indigenous and 427 non-Indigenous multiples births

^a^Infant mortality rate: deaths under 12 months, per 1000 live births

^b^Prison before, but not during, pregnancy

In non-Indigenous infants, those whose mothers were imprisoned before (9.4 per 1000) or during (8.2 per 1000) pregnancy had apparently lower rates of infant mortality than those whose mothers were imprisoned for the first time after their birth (14.2 per 1000) or who had community orders alone (12.6 per 1000). This difference was not significant and confidence intervals wide, possibly due to the low numbers of infant deaths within the sample of non-Indigenous children with mothers imprisoned before or during pregnancy.

### Univariate analysis of infant mortality

As shown in Table 2, abruptio placentae and other placental disorders (excluding placenta previa) were associated with the highest risk of death for both Indigenous and non-Indigenous infants. Other important risk factors for Indigenous and non-Indigenous infants were low socioeconomic status, multiple gestation pregnancies, birth spacing < 18-months, having an older sibling in contact with the child protection system during an infant’s gestation, and maternal substance use or poisoning-related service contacts during pregnancy.

**Table 2:** Risk factors for infant mortality, by Indigenous status

	Indigenous infants (*n* = 25,962)						Non-Indigenous infants (*n* = 16,712)					
	Survived	Died					Survived	Died				
	*n*	*n*	%	RR	(95% CI)	*p*-value	*n*	*n*	%	RR	(95% CI)	*p*-value
Infants	25,532	430	1.7				16,596	116	0.7			
Demographic factors												
Sex^a^												
Male	13,052	240	1.8	1.20	(0.99–1.45)	0.058	8586	64	0.7	1.14	(0.79–1.65)	0.468
Female*	12,438	190	1.5				7991	52	0.6			
Maternal Indigenous status												
Indigenous	24,321	418	1.7	1.72	(0.97–3.05)	0.062						
Non-Indigenous*	1211	12	1.0				16,596	116	0.7			
Socioeconomic status^b^												
Very low (0–5%)	6793	128	1.8	2.16	(1.38–3.39)	0.001	996	15	1.5	3.50	(1.88–6.54)	<.001
Low (6–25%)	11,095	195	1.7	2.02	(1.30–3.13)	0.002	4461	50	1.1	2.62	(1.65–4.15)	<.001
Medium (26–50%)	5054	83	1.6	1.89	(1.18–3.01)	0.008	4517	23	0.5	1.20	(0.69–2.07)	0.523
High (51–100%)*	2546	22	0.9				6584	28	0.4			
Geographical remoteness^c^												
Major cities/Inner regional*	10,331	160	1.5				13,574	88	0.6			
Outer regional	4185	73	1.7	1.12	(0.85–1.48)	0.404	1811	22	1.2	1.86	(1.17–2.97)	0.009
Remote	4776	75	1.5	1.01	(0.77–1.33)	0.922	834	5	0.6	0.93	(0.38–2.27)	0.865
Very remote	6195	120	1.9	1.25	(0.98–1.58)	0.066	326	< 5	0.3	0.47	(0.07–3.40)	0.458
Baseline Pregnancy Risk Factors												
Multiple gestation												
Yes	568	23	3.9	2.43	(1.61–3.66)	<.001	414	13	3.0	4.81	(2.73–8.50)	<.001
No*	24,964	407	1.6				16,182	103	0.6			
Birth spacing												
< 18 months	3011	82	2.7	1.74	(1.37–2.21)	<.001	1112	19	1.7	2.70	(1.66–4.40)	<.001
Firstborn/18 months+*	22,521	348	1.5				15,484	97	0.6			
Maternal age												
12–19 years	7628	122	1.6	0.91	(0.72–1.17)	0.471	1740	21	1.2	2.36	(1.41–3.94)	0.001
20–24 years	9420	158	1.6	0.96	(0.76–1.20)	0.713	3916	47	1.2	2.35	(1.57–3.52)	<.001
25–34 years*	7533	132	1.7				9054	46	0.5			
35 + years	951	18	1.9	1.08	(0.66–1.76)	0.761	1886	< 5	0.1	0.21	(0.05–0.86)	0.030
Parity												
Nulliparous	9035	126	1.4	0.82	(0.66–1.03)	0.093	7954	53	0.7	0.99	(0.67–1.45)	0.939
Parity 1–2*	10,847	184	1.7				7539	51	0.7			
Parity 3+	5650	120	2.1	1.25	(0.99–1.57)	0.058	1103	12	1.1	1.60	(0.86–2.99)	0.140
Pregnancy complications												
Abruptio placentae and other disorders^d^												
Yes	122	8	6.2	3.77	(1.91–7.42)	<.001	90	< 5	4.3	6.31	(2.38–16.8)	<.001
No*	25,410	422	1.6				16,506	112	0.7			
Placenta previa												
Yes	87	< 5	3.3	2.02	(0.66–6.17)	0.217	109	0	0			
No*	25,445	427	1.7				16,487	116	0.7			
Premature rupture of membranes												
Yes	1200	34	2.8	1.72	(1.22–2.43)	0.002	445	5	1.1	1.63	(0.67–3.97)	0.284
No*	24,332	396	1.6				16,151	111	0.7			
Infection related hospitalisation in pregnancy^e^												
Yes	2041	34	1.6	0.99	(0.70–1.40)	0.947	388	< 5	0.5	0.73	(0.18–2.96)	0.664
No*	23,491	396	1.7				16,208	114	0.7			
Other maternal factors/Exposures during pregnancy												
Substance use related service contact^f^												
Yes	397	15	3.6	2.24	(1.35–3.72)	0.002	293	6	2.0	2.99	(1.33–6.76)	0.008
No*	25,135	415	1.6				16,303	110	0.7			
Hospitalisation for external causes of injury^g^												
Yes	1479	38	2.5	1.56	(1.12–2.17)	0.008	278	< 5	1.1	1.55	(0.50–4.86)	0.450
No*	24,053	392	1.6				16,318	113	0.7			
Mental health related service contact^h^												
Yes	768	12	1.5	0.93	(0.52–1.64)	0.794	427	5	1.2	1.70	(0.70–4.14)	0.244
No*	24,764	418	1.7				16,169	111	0.7			
Sibling in contact with child protection^i^												
Yes	852	30	3.4	2.13	(1.48–3.07)	<.001	237	6	2.5	3.70	(1.64–8.33)	0.002
Firstborn/No*	24,680	400	1.6				16,359	110	0.7			

*Reference category

^a^42 Indigenous and 19 non-Indigenous infants missing sex

^b^46 Indigenous and 38 non-Indigenous infants missing socioeconomic status

^c^47 Indigenous and 51 non-Indigenous infants missing remoteness

^d^Excludes placenta previa

^e^Infection related hospitalisation in pregnancy

^f^Substance use (including alcohol) or poisoning related service contact (hospital or mental health service)

^g^Hospitalisation for external causes of injury in pregnancy (excludes poisoning)

^h^Mental health service contact in pregnancy (excludes substance use) (hospital or mental health service)

^i^Older sibling(s) in contact with child protection system in infant’s pregnancy

Maternal hospitalisations for premature rupture of membranes, or external causes of injury were important risk factors for infant mortality only for Indigenous infants. Male sex was also only a risk factor for infants in the Indigenous subgroup. Young maternal age and geographical location for those living in outer regional areas, were only significant risk factors in the non-Indigenous sample.

Factors not strongly associated with infant death included parity, infection related hospitalisations, or mental health related service contact (not related to substance use) in pregnancy, and hospital admissions for placenta previa.

### Multivariate regression of indigenous infant mortality

Multivariate regression was only performed for Indigenous children because of the low number of deaths in the sample of non-Indigenous infants with incarcerated mothers.

Variables for maternal Indigenous status, geographical remoteness, maternal age, parity, placenta previa, infection-related hospitalisations, external injury-related hospitalisations, and mental health related service contacts in pregnancy (other than for substance use) were excluded based on their lack of association with infant mortality in our data (Additional file 2). The remaining variables were included and are shown in Model 1 (Table 3). In Models 2–4, variables were removed by group in order of; other maternal factors and exposures (Model 2), pregnancy complications (Model 3), baseline pregnancy risk factors (Model 4), leaving the combined effects of maternal corrections history and demographic factors on infant mortality in Model 4.

**Table 3:** Regression model of infant mortality, Indigenous children

	Model 1			Model 2			Model 3			Model 4		
	RR	(95% CI)	*p*-value	RR	(95% CI)	*p*-value	RR	(95% CI)	*p*-value	RR	(95% CI)	*p*-value
Maternal corrections history												
Prison (before pregnancy*)	1.83	(1.36–2.47)	<.001	1.99	(1.49–2.67)	<.001	2.03	(1.51–2.71)	<.001	2.10	(1.57–2.81)	<.001
Prison (during pregnancy)	2.55	(1.69–3.86)	<.001	2.96	(1.98–4.43)	<.001	3.01	(2.01–4.51)	<.001	3.10	(2.07–4.64)	<.001
Prison (after birth only)	1.55	(1.18–2.03)	0.001	1.64	(1.25–2.14)	<.001	1.67	(1.28–2.18)	<.001	1.73	(1.32–2.25)	<.001
Community-only	1.38	(1.07–1.77)	0.012	1.41	(1.10–1.81)	0.007	1.42	(1.11–1.83)	0.006	1.45	(1.13–1.87)	0.003
No corrections history	ref.											
Demographic factors												
Birth year	0.96	(0.95–0.97)	<.001	0.96	(0.95–0.98)	<.001	0.96	(0.95–0.98)	<.001	0.97	(0.95–0.98)	<.001
Sex												
Male	1.21	(1.01–1.47)	0.044	1.22	(1.01–1.47)	0.039	1.22	(1.01–1.47)	0.038	1.22	(1.01–1.48)	0.036
Female	ref.											
Socioeconomic status												
Very low (0–5%)	2.11	(1.35–3.31)	0.001	2.08	(1.33–3.27)	0.001	2.1	(1.34–3.30)	0.001	2.11	(1.34–3.31)	0.001
Low (6–25%)	1.88	(1.21–2.91)	0.005	1.86	(1.20–2.88)	0.006	1.87	(1.20–2.89)	0.005	1.91	(1.23–2.96)	0.004
Medium (26–50%)	1.75	(1.10–2.80)	0.018	1.74	(1.09–2.78)	0.02	1.75	(1.10–2.79)	0.019	1.78	(1.11–2.83)	0.016
High (51–100%)	ref.											
Baseline pregnancy risk factors												
Multiple gestation												
Yes	2.29	(1.51–3.46)	<.001	2.37	(1.57–3.59)	<.001	2.58	(1.71–3.88)	<.001			
No	ref.											
Birth spacing												
< 18 months	1.52	(1.19–1.93)	0.001	1.57	(1.24–2.00)	<.001	1.60	(1.26–2.03)	<.001			
Firstborn/18 months+	ref.											
Pregnancy complications												
Abruptio placentae and other disorders^a^												
Yes	2.85	(1.46–5.59)	0.002	2.92	(1.49–5.72)	0.002						
No	ref.											
Premature rupture of membranes												
Yes	1.66	(1.18–2.35)	0.004	1.67	(1.18–2.37)	0.003						
No	ref.											
Other maternal factors/exposures in pregnancy												
Substance use related service contact												
Yes	1.71	(1.02–2.87)	0.042									
No	ref.											
External injury related hospitalisation												
Yes	1.20	(0.86–1.68)	0.283									
No	ref.											
Sibling with Child Protection contact												
Yes	1.57	(1.07–2.31)	0.022									
Firstborn/No	ref.											
Observations	25,875			25,875			25,875			265,875		
AIC	0.1640			0.1642			0.1646			0.1656		

*Prison before, but not during, pregnancy

^a^Excludes placenta previa

The best model fit was achieved with the inclusion of all variables (i.e., Model 1). Abruptio placentae and other placental disorders contributed the highest risk of infant death, followed by maternal imprisonment during pregnancy, and multiple gestation pregnancy. The effect of birth year remained stable across Models 1–4 and approximated to a 4% reduction in risk of infant mortality each year.

### Prevalence of key demographic and pregnancy risk factors by maternal corrections history

Table 4 shows the prevalence of pregnancy risk factors selected from the previous univariate and multivariate analyses, for Indigenous and non-Indigenous infants reported by their mothers’ contact with corrective services.

**Table 4:** Prevalence of pregnancy risk factors for infant mortality, by maternal corrections exposure and Indigenous status

	Indigenous infants					Non-Indigenous infants				
	Prison (before preg.)	Prison (during preg.)	Prison (after birth)	Community correct.	No correct.	Prison (before preg.)	Prison (during preg.)	Prison (after birth)	Community correct	No correct
	*n* = 2857	*n* = 802	*n* = 3658	*n* = 5828	*n* = 12,817	*n* = 667	*n* = 249	*n* = 2588	*n* = 653	*n* = 12,555
Very low (0–5%) SES^a^										
%	22.9	22.7	29.1	29.8	25.7	13.3	13.4	12.8	12.3	3.8
RR	0.89	0.88	1.13	1.16	ref.	3.48	3.49	3.35	3.20	ref.
*p-*value	0.002	0.057	<.001	<.001		<.001	<.001	<.001	<.001	
Multiple gestation										
%	1.9	3.0	2.5	2.5	2.1	4.0	1.6	2.4	2.6	2.5
RR	0.86	1.39	1.18	1.17	ref.	1.60	0.63	0.93	1.03	ref.
*p-*value	0.328	0.113	0.152	0.126		0.017	0.362	0.602	0.911	
Birth spacing < 18mths										
%	17.9	11.2	14.1	12.8	9.6	12.7	8.0	9.8	10.4	5.6
RR	1.87	1.17	1.47	1.34	ref.	2.27	1.43	1.75	1.86	ref.
*p-*value	<.001	0.127	<.001	<.001		<.001	0.099	<.001	<.001	
Maternal age < 20 yrs.										
%	15.3	28.6	41.7	33.1	28.3	9.3	10	25.9	26	6.6
RR	0.54	1.01	1.47	1.17	ref.	1.40	1.51	3.91	3.92	ref.
*p-*value	<.001	0.895	<.001	<.001		0.007	0.032	<.001	<.001	
Placental disorders^b^										
%	0.5	0.7	0.6	0.7	0.4	0.1	0.8	1.1	1.1	0.4
RR	1.17	1.92	1.54	1.72	ref.	0.34	1.83	2.56	2.45	ref.
*p-*value	0.620	0.130	0.090	0.011		0.288	0.398	<.001	0.025	
PROM^c^										
%	6.8	5.5	5.1	5.2	4.0	5.5	3.2	3.7	3.2	2.3
RR	1.71	1.39	1.28	1.32	ref.	2.41	1.4	1.59	1.40	ref.
*p-*value	<.001	0.032	0.003	<.001		<.001	0.344	<.001	0.133	
Substance use^d^										
%	3.2	7.4	2.2	1.6	0.7	12.3	10.0	4.7	3.2	0.4
RR	4.64	10.59	3.15	2.27	ref.	30.87	25.21	11.74	8.08	ref.
*p-*value	<.001	<.001	<.001	<.001		<.001	<.001	<.001	<.001	
External injury^e^										
%	10.9	14.5	9.4	6.5	2.9	6.9	6.4	3.4	2.1	0.9
RR	3.78	5.02	3.26	2.25	ref.	7.46	6.95	3.72	2.32	ref.
*p-*value	<.001	<.001	<.001	<.001		<.001	<.001	<.001	0.003	
Mental health contact^f^										
%	6.7	8.4	3.6	3.1	1.6	13.5	10.8	5.0	5.1	1.2
RR	4.20	5.22	2.27	1.96	ref.	11.15	8.96	4.15	4.17	ref.
*p-*value	<.001	<.001	<.001	<.001		<.001	<.001	<.001	<.001	
Sibling in contact CP^g^										
%	11.6	14.3	5.2	2.6	0.7	12.9	11.2	4.4	1.5	0.05
RR	15.68	19.35	6.97	3.50	ref.	269.80	235.30	91.36	32.04	ref.
*p-*value	<.001	<.001	<.001	<.001		<.001	<.001	<.001	<.001	

^a^46 Indigenous and 38 non-Indigenous infants missing socioeconomic status

^b^Excludes placenta previa

^c^Premature rupture of membranes

^d^Substance use (including alcohol) or poisoning related service contact (Mental Health or Hospital)

^e^Hospitalisation for external causes of injury in pregnancy (excludes poisoning)

^f^Mental health related service contact in pregnancy (excludes substance use) (Mental Health or Hospital)

^g^Older sibling(s) in contact with child protection system in infant’s pregnancy

Infants of mothers with any corrections history had, in most instances, higher prevalence of maternal service contact in pregnancy for substance use (including poisoning), external causes of injury, mental health related service contacts, and sibling contact with the child protection system compared to infants whose mothers had no record with corrective services. Prevalence of these service contacts in pregnancy was highest for infants whose mothers were imprisoned during pregnancy, and higher for infants whose mothers were imprisoned before pregnancy compared to those imprisoned for the first time after birth or who had community corrections orders for these service contacts.

The proportion of infants born in areas of very low socioeconomic status was high for all groups of Indigenous children (> 20%) and did not differ by maternal corrections history. There was a higher proportion of low socioeconomic status for non-Indigenous children with any maternal corrections record (12–13%) compared to those with no maternal corrections history (4%).

There was a higher prevalence of a birth spacing of less than 18-months for infants with any maternal corrections record compared to no maternal corrections history, except the difference was not significant for those infants whose mothers were imprisoned during pregnancy. The prevalence of infants born to mothers aged less than 20 years was highest among infants whose mothers were first imprisoned after birth or had community-corrections orders.

For Indigenous children, maternal hospitalisation for abruptio placentae and other placental disorders (excluding placenta previa) was not different between infants by maternal corrections history, whereas prevalence of maternal hospitalisation for premature rupture of membranes was higher where there had been any record of maternal contact with the corrections system.

For the non-Indigenous children, hospitalisation of the mother for abruptio placentae and other placental disorders (excluding placenta previa) was higher where infants’ mothers were first imprisoned after birth or had community-correctional orders, and prevalence of hospitalisation for premature rupture of membranes was higher where infants’ mothers were imprisoned before or after pregnancy.

## Discussion

This is the first study to provide a comprehensive investigation of infant mortality outcomes for children of women prisoners. Children of mothers with a history of contact with corrective services, including community-based corrections orders and imprisonment before or after pregnancy, had increased rates of infant mortality.

Within the Indigenous sample, rates of infant mortality were highest for infants whose mothers were imprisoned during pregnancy, when compared to similarly disadvantaged mothers who were imprisoned at times other than pregnancy or who had community-based correctional orders. The strength of the relationship between infant mortality and maternal imprisonment in pregnancy for Indigenous infants remained in the full model after adjusting for other important risk factors. The only determinant to have a greater association with Indigenous infant mortality was abruptio placentae and other placental disorders, a serious pregnancy complication. Imprisonment during pregnancy was a stronger determinant of infant mortality than all other pregnancy complications and baseline pregnancy risk factors including multiple gestation pregnancies. Indigenous infants whose mothers were imprisoned during pregnancy also experienced the highest prevalence of maternal contact with services during pregnancy for substance use, mental illness, and external injury. These findings clearly demonstrate the significant vulnerability of Indigenous infants whose mothers are imprisoned during pregnancy.

It was not possible to determine the relationship between maternal imprisonment before and during pregnancy and the risk of infant mortality for non-Indigenous infants due to the relatively small numbers of infants in the sample populations whose mothers were imprisoned before or during pregnancy. However, non-Indigenous infants whose mothers who were imprisoned before or during pregnancy had a significantly higher prevalence of several pregnancy risk factors including maternal service contact in pregnancy for substance use, external injury, or mental health issues, and having siblings in contact with the child protection system, compared to non-Indigenous infants whose mothers were first imprisoned after birth or had community-based corrections orders alone.

Infant mortality is a marker of adversity which is strongly linked to social and economic disadvantage [10]. It is well-established that Indigenous children experience higher rates of socioeconomic disadvantage and infant mortality than non-Indigenous children [10, 29]. Over a quarter of our Indigenous subgroup, compared to only 6% of the non-Indigenous sample, was born in the lowest 5% of areas by socioeconomic status. Socioeconomic disadvantage provides a broad measure of social determinants such as parental education, employment, disability, and overcrowding as well as a greater prevalence of health conditions and risk behaviours such as alcohol and substance use, domestic violence, and mental illness, which are related to infant mortality risk. For Indigenous peoples, socioeconomic disadvantage is also associated with experiences of racism and discrimination in service access and broader society, and in increased contact with health and criminal justice systems [30].

It is recognised that imprisonment likely acts as both a proxy for socioeconomic disadvantage and for risk behaviours which are associated with imprisonment, including increased substance use, injury and mental illness, as evidenced in our study by increased prevalence of service contacts related to these risks in pregnancy for pregnant prisoners. Similarly, having a sibling in contact with the child protection system during pregnancy can be considered as a proxy for maternal vulnerability and socioeconomic disadvantage. Whether maternal imprisonment during pregnancy has an impact on birth outcomes, including infant mortality, over-and-above the effects of pre-existing disadvantage, is a key issue within the international literature [3].

It has been proposed that there may be a possible protective effect of imprisonment in pregnancy for birth outcomes [3]. The proposed protective effect of imprisonment in pregnancy on birth outcomes is thought to be contributed to by a reduction of exposure to risk factors such as domestic violence and substance use while in custody, and in improved nutrition and access to antenatal care. Within our study, infants whose mothers were imprisoned before pregnancy had a higher prevalence of risk factors during pregnancy related to maternal service contact for substance use, injury, and mental illness, when compared to infants whose mothers were first imprisoned after birth. This finding suggests that risk behaviours of this kind do occur concurrently with imprisonment. However, the strength of our findings with respect to maternal imprisonment during pregnancy for Indigenous infants suggest there may be an additional impact of imprisonment during pregnancy on infant mortality risk, at least in certain circumstances.

Similar to our findings, the general protective effect of imprisonment during pregnancy on birth outcomes that has been reported in the broader international literature was not replicated in the only prior Australian study of the effects of maternal imprisonment on pregnancy outcomes [4]. While just over one-quarter of pregnant prisoners were Indigenous, Walker and colleagues [4] did not investigate the outcomes for the Indigenous and non-Indigenous populations separately. Our study has clearly demonstrated that infant mortality is higher for Western Australian Indigenous infants whose mothers were imprisoned during pregnancy. Taken together, our study and that of Walker and colleagues [4] suggest that there are different outcomes for the infants of Australian women imprisoned during pregnancy than those reported for other jurisdictions [3]. It is not yet clear, however, whether this difference is restricted to the Indigenous population as our findings were inconclusive, due to small numbers, with respect to non-Indigenous infants.

The difference in outcomes for Australian pregnant prisoners compared to those within other criminal justice systems reported in the international literature [3], may relate to the longer periods of imprisonment experienced by women in other jurisdictions compared to Australia, as an increased length of imprisonment in pregnancy is associated with higher birth weight which represents a positive pregnancy outcome [31, 32]. For example, Walker and colleagues [4] reported the average length of stay for sentenced women prisoners in New South Wales, Australia, is 196 days compared to 547 days in US prisons [4]. In our study of Western Australian children exposed to maternal incarceration before their second birthday, almost half of all prison stays for either sentenced prisoners or unsentenced remandees were < 2 weeks [33]. In this context the presumption that imprisonment has a ‘dose-response’ effect on perinatal outcomes [32], and that short-term imprisonment does not carry excess risks for the mother or her infant warrants further research.

Within Western Australia, all pregnant prisoners are provided with health care “commensurate with community standards” [34]. However, health service provision varies between prisons [35–40], and regional prisons face additional challenges such as in the transportation of prisoners to community health centres [41]. There is limited evidence available on the provision and impact of antenatal care provided to pregnant prisoners in Australia. In New South Wales, Walker and colleagues [4] found that women imprisoned during pregnancy were more likely to initiate antenatal care after 20 weeks gestation than women with no record of imprisonment. However as many of these women were not imprisoned for the duration of pregnancy, as was the case for women in the present study, it is possible antenatal care was first initiated during imprisonment.

Imprisonment during pregnancy may have negative impacts on the mental health and wellbeing of women, particularly for Indigenous mothers, as a consequence of being separated from family and country during their pregnancy [42]. There is evidence that maternal stress in pregnancy can impact on birth outcomes [43, 44]. Research on offender health has demonstrated that the year following release from prison, particularly within the first month of release, is also a key risk period for an offender’s own hospitalisation and mortality [45–48]. Whether the release period also leads to increased risk for offenders’ children, including the unborn children of pregnant prisoners, has not been investigated. The results from the present study highlight that this as an important area of future research.

Further research is needed to understand whether there are particular characteristics of maternal imprisonment during pregnancy that are associated with infant mortality, and whether the effect is restricted to Indigenous populations. The study findings highlight the importance of separate consideration of Indigenous populations when investigating outcomes for children of prisoners, as combining populations may mask important differences in outcomes.

### Limitations

These results need to be considered within the context of the study’s limitations, for example, the power to detect relationships between infant mortality and maternal imprisonment before or during pregnancy in the non-Indigenous sample was limited by the small numbers of non-Indigenous infant deaths reported within those groups.

As gestational age was not available, pregnancy was taken to begin nine-months prior to month of birth for all infants. Accordingly, some records of exposure to maternal imprisonment, and service contacts related to substance use, mental health or child protection, that were attributed to having occurred during pregnancy may have occurred prior to pregnancy if gestation was shorter than nine-months. There is evidence, however, that adverse events within the preconception period (6–0 months before pregnancy) can increase infant mortality risk [49, 50]. Therefore any records of exposure misclassified as having occurred during pregnancy, may still have been expected to have an impact on infant mortality risk.

Additionally, without gestational age we have not been able to measure preterm birth (delivery before 37 weeks of gestation), which is associated with infant mortality [51]. However, preterm birth shares many of the same risk factors for infant mortality identified within the study including low socioeconomic status, maternal age, maternal stress, infections, and multiple gestation pregnancies [51]. The study has provided the first evidence of an association between maternal incarceration in pregnancy and infant mortality, further research is needed investigate the impact of factors not able to be measured in the current study, notably preterm birth, antenatal care and caesarean section rates.

Administrative data alone cannot fully capture occurrences of heavy drinking or substance use, mental illness, or injuries resulting from domestic violence, within pregnancy [52]. In addition, the study only obtained the primary diagnosis code (not co-diagnoses) for hospital and mental health service records. Consequently, the associations observed in our study with drinking or other substance use in pregnancy, mental illness or injuries are likely under ascertained.

Hospital data were not obtained for second-generation children. While explanatory variables based on hospital data were primarily taken from maternal hospital records (available for all mothers), there may have been some missed cases of chromosomal abnormalities, and substance use related service contacts in pregnancy, for the second-generation children.

## Conclusions

To date there have been few studies which have focussed on the impact of maternal imprisonment on infant mortality. This study provides the first detailed analysis of infant mortality outcomes for children whose mothers were imprisoned in pregnancy. The study demonstrates that there are higher rates of infant mortality for Indigenous, compared to non-Indigenous, children of prisoners and that within the Indigenous sample any maternal contact with the corrections system is associated with an increase in infant mortality.

Maternal imprisonment in pregnancy is an important determinant of infant mortality for Indigenous children. Further research is needed to determine what factors contribute to this increased risk of infant mortality, and whether particular groups of prisoners are more affected. Due to the relatively low incidence of infant deaths within the non-Indigenous sample, it was not possible to determine the impact of maternal imprisonment on infant mortality in this sub-population. It was the case, however, that non-Indigenous infants whose mothers were imprisoned before or during pregnancy experienced higher rates of pregnancy risk factors, than infants whose mothers were first imprisoned after birth or had community-based correctional orders. This highlights the vulnerability of non-Indigenous and Indigenous pregnant prisoners, and the importance of providing support services to address pregnancy risk factors for women in contact with the corrections system.

## Additional files

Additional file 1: ICD codes. List of ICD codes used to define the study variables. (PDF 393 kb)

Additional file 2: Regression models of infant mortality by main group for Indigenous children. Results of the multivariate regression conducted for each main grouping of variables to eliminate non-significant variables from including the final multivariate analysis. (PDF 330 kb)

## Abbreviations

AIC: Akaike Information Criterion
ARIA: Accessibility/Remoteness Index of Australia
CPFS: Department of Communities: Child Protection and Family Support
HMDC: Hospital Morbidity Data System Collection
ICD: International Classification of Diseases
MHIS: Mental Health Information System
SEIFA: Socio-economic Indexes for Areas
WADLS: Western Australian Data Linkage System
