# Supplementary material for: Determinants of infant mortality for children of women prisoners: a longitudinal linked data study
Source: BMC Pregnancy Childbirth. 2018 Jun 1;18:202. doi: 10.1186/s12884-018-1840-z (PMC5984779; doi:10.1186/s12884-018-1840-z)
Supplement: Supplementary file 1 — ICD codes. List of ICD codes used to define the study variables. (PDF 393 kb) [file 12884_2018_1840_MOESM1_ESM.pdf]

EXPOSURE TO ALCOHOL, DRUGS AND OTHER TOXINS IN PREGNANCY

Chapter / Diagnosis level 1	Diagnosis level 2	ICD ed.	ICD codes
**Endocrine, nutritional and metabolic diseases, etc.**			
Diseases of other endocrine glands	Alcoholic pseudo-Cushing syndrome	9	255.0
**Mental disorders**			
Organic psychotic conditions	Alcoholic psychoses	9	291
Organic psychotic conditions	Drug psychoses	9	292
Psychoactive substance	Alcohol dependence syndrome	9	303
Psychoactive substance	Drug dependence	9	304
Psychoactive substance	Nondependent abuse of drugs	9	305
**Diseases of the nervous system**			
Degenerative diseases of the central nervous system	Alcoholic nervous system degeneration	9	331.7
Disorders of the peripheral nervous system	Alcoholic polyneuropathy	9	357.5
Disorders of the peripheral nervous system	Toxic myopathy	9	359.4
**Diseases of the circulatory system**			
Other forms of heart disease	Alcoholic cardiomyopathy	9	425.5
**Diseases of the digestive system**			
Diseases of esophagus, stomach, and duodenum	Alcoholic gastritis	9	535.3
Other diseases of digestive system	Alcoholic chronic liver disease	9	571.0-571.3
Other diseases of digestive system	Alcoholic pancreatitis	9	577.1
**Complications of pregnancy, childbirth, and the puerperium**			
Complications mainly related to pregnancy	Drug dependence complicating pregnancy, etc.	9	648.3-648.34
Complications mainly related to pregnancy	Tobacco use disorder complicating pregnancy, etc.	9	649
Normal delivery, other indications for care in pregnancy	Known or suspected fetal abnormality, alcohol	9	655.43
**Certain conditions originating in the perinatal period**			
Maternal causes of perinatal morbidity and mortality	Noxious influences affecting fetus or newborn	9	760.7
Maternal causes of perinatal morbidity and mortality	Fetal alcohol syndrome	9	760.71
Maternal causes of perinatal morbidity and mortality	Exposure to narcotics, perinatal	9	760.72
Maternal causes of perinatal morbidity and mortality	Other noxious influences affecting fetus/newborn	9	760.79
Ill-defined conditions originating in the perinatal period	Drug reactions/intoxications specific to newborn	9	779.4
Ill-defined conditions originating in the perinatal period	Drug withdrawal syndrome in newborn	9	779.5
**Symptoms, signs, and ill-defined conditions**			
Nonspecific abnormal findings	Excessive blood level of alcohol	9	790.3
**Injury and poisoning**			
Poisoning by drugs, medicinal and biological substances	by opium	9	965.0-965.00
Poisoning by drugs, medicinal and biological substances	by heroin	9	965.01
Poisoning by drugs, medicinal and biological substances	by methadone	9	965.02
Poisoning by drugs, medicinal and biological substances	by other opiates and related narcotics	9	965.09
Poisoning by drugs, medicinal and biological substances	by barbiturates	9	967.0
Poisoning by drugs, medicinal and biological substances	by chloral hydrate group	9	967.1
Poisoning by drugs, medicinal and biological substances	by paraldehyde	9	967.2
Poisoning by drugs, medicinal and biological substances	by bromine compounds	9	967.3
Poisoning by drugs, medicinal and biological substances	by methaqualone compounds	9	967.4
Poisoning by drugs, medicinal and biological substances	by glutethimide group	9	967.5
Poisoning by drugs, medicinal and biological substances	by mixed sedatives	9	967.6
Poisoning by drugs, medicinal and biological substances	by other sedatives and hypnotics	9	967.8
Poisoning by drugs, medicinal and biological substances	by unspecified sedative or hypnotic	9	967.9
Poisoning by drugs, medicinal and biological substances	by antidepressants	9	969.0
Poisoning by drugs, medicinal and biological substances	by phenothiazine-based tranquilizers	9	969.1
Poisoning by drugs, medicinal and biological substances	by butyrophenone-based tranquilizers	9	969.2
Poisoning by drugs, medicinal and biological substances	by antipsychotics, neuroleptics, major tranquilizers	9	969.3
Poisoning by drugs, medicinal and biological substances	by benzodiazepine-based tranquilizers	9	969.4
Poisoning by drugs, medicinal and biological substances	by other tranquilizers	9	969.5
Poisoning by drugs, medicinal and biological substances	by psychodysleptics (hallucinogens)	9	969.6
Poisoning by drugs, medicinal and biological substances	by psychostimulants	9	969.7
Poisoning by drugs, medicinal and biological substances	by other specified psychotropic agents	9	969.8
Poisoning by drugs, medicinal and biological substances	by unspecified psychotropic agents	9	969.9
Poisoning by drugs, medicinal and biological substances	by opiate antagonists	9	970.1
Poisoning by drugs, medicinal and biological substances	by other specified CNS stimulants (inc. cocaine)	9	970.8
Poisoning by drugs, medicinal and biological substances	by unspecified CNS stimulants	9	970.9
Poisoning by drugs, medicinal and biological substances	by alcohol deterrents	9	977.3
Toxic effects of substances chiefly nonmedicinal	Toxic effect of alcohol	9	980
Toxic effects of substances chiefly nonmedicinal	Toxic effect of petroleum products	9	981
Toxic effects of substances chiefly nonmedicinal	Toxic effect of other solvents	9	982
Toxic effects of substances chiefly nonmedicinal	Toxic effect of lead (including fumes)	9	984
Toxic effects of substances chiefly nonmedicinal	Toxic effect of other gases, fumes, or vapors	9	987

**External causes of injury**			
Accidental poisoning by drugs, medicinal substances, etc.	by heroin	9	E850.0
Accidental poisoning by drugs, medicinal substances, etc.	by methadone	9	E850.1
Accidental poisoning by drugs, medicinal substances, etc.	by other opiates and related narcotics	9	E850.2
Accidental poisoning by drugs, medicinal substances, etc.	by barbiturates	9	E851
Accidental poisoning by drugs, medicinal substances, etc.	by sedatives and hypnotics	9	E852
Accidental poisoning by drugs, medicinal substances, etc.	by tranquilizers	9	E853
Accidental poisoning by drugs, medicinal substances, etc.	by other psychotropic agents	9	E854
Accidental poisoning by other solid and liquid substances	by alcohol not elsewhere	9	E860
Accidental poisoning by other solid and liquid substances	by petroleum, other solvents	9	E862
Accidental poisoning by other solid and liquid substances	by other gases and vapors	9	E869
Suicide and self-inflicted injury	Suicide and self-inflicted poisoning solid, liquid	9	E950
Suicide and self-inflicted injury	Suicide and self-inflicted poisoning by dgases	9	E951
Suicide and self-inflicted injury	Suicide and self-inflicted poisoning by other gases	9	E952
Undetermined Whether Accidentally Or Purposely Inflicted	by analgesics, antipyretics, and antirheumatics	9	E980.0
Undetermined Whether Accidentally Or Purposely Inflicted	by barbiturates	9	E980.1
Undetermined Whether Accidentally Or Purposely Inflicted	other sedatives and hypnotics	9	E980.2
Undetermined Whether Accidentally Or Purposely Inflicted	by tranquilizers and other psychotropic agents	9	E980.3
Undetermined Whether Accidentally Or Purposely Inflicted	by other specified drugs and medicinal substances	9	E980.4
Undetermined Whether Accidentally Or Purposely Inflicted	by unspecified drug or medicinal substance	9	E980.5
Undetermined Whether Accidentally Or Purposely Inflicted	by other and unspecified solid, liquid substances	9	E980.9
Undetermined Whether Accidentally Or Purposely Inflicted	Poisoning by gases in domestic use	9	E981
Undetermined Whether Accidentally Or Purposely Inflicted	Poisoning by other gases	9	E982
**Supplementary classification of factors**			
Persons with potential health hazards	Personal history of alcoholism	9	V11.3
Persons encountering health services, other circumstances	Counseling on substance use and abuse	9	V65.42
Special screening for mental disorders	Screening for alcoholism	9	V79.1
**Endocrine, nutritional and metabolic diseases**			
Disorders of other endocrine glands	Drug-induced Cushing syndrome	10	E24.2
Disorders of other endocrine glands	Alcohol-induced pseudo-Cushing syndrome	10	E24.4
**Mental and behavioural disorders**			
Due to psychoactive substance use	due to use of alcohol	10	F10
Due to psychoactive substance use	due to use of opioids	10	F11
Due to psychoactive substance use	due to use of cannabinoids	10	F12
Due to psychoactive substance use	due to use of sedatives or hypnotics	10	F13
Due to psychoactive substance use	due to use of cocaine	10	F14
Due to psychoactive substance use	due to use of other stimulants, including caffeine	10	F15
Due to psychoactive substance use	due to use of hallucinogens	10	F16
Due to psychoactive substance use	due to use of tobacco	10	F17
Due to psychoactive substance use	due to use of volatile solvents	10	F18
Due to psychoactive substance use	due to multiple drug use, or other substances	10	F19
**Diseases of the nervous system**			
Other degenerative diseases of the nervous system	Degeneration of nervous system due to alcohol	10	G31.2
Polyneuropathies, disorders of peripheral nervous system	Drug-induced polyneuropathy	10	G62.0
Polyneuropathies, disorders of peripheral nervous system	Alcoholic polyneuropathy	10	G62.1
Diseases of myoneural junction and muscle	Drug-induced myopathy	10	G72.0
Diseases of myoneural junction and muscle	Alcoholic myopathy	10	G72.1
**Diseases of the circulatory system**			
Other forms of heart disease	Alcoholic cardiomyopathy	10	I42.6
Other forms of heart disease	Cardiomyopathy due to drugs and external agents	10	I42.7
**Diseases of the digestive system**			
Diseases of oesophagus, stomach and duodenum	Alcoholic gastritis	10	K29.2
Diseases of liver	Alcoholic liver disease	10	K70
Disorders of gallbladder, biliary tract and pancreas	Alcohol-induced chronic pancreatitis	10	K86.0
**Pregnancy, childbirth and the puerperium**			
Maternal care for known/suspected fetal abnorm/damage	Maternal care for damage to fetus from alcohol	10	O35.4
Maternal care for known/suspected fetal abnorm/damage	Maternal care for damage to fetus by drugs	10	O35.5
**Certain conditions originating in the perinatal period**			
Fetus/newborn affected noxious via placenta/breast milk	Fetus/newborn affected by maternal use tobacco	10	P04.2
Fetus/newborn affected noxious via placenta/breast milk	Fetus/newborn affected by maternal use alcohol	10	P04.3
Fetus/newborn affected noxious via placenta/breast milk	Fetus/newborn affected by maternal use drugs	10	P04.4
**Congenital malformations, deformations, etc.**			
Other congenital malformations	Fetal alcohol syndrome (dysmorphic)	10	Q86.0
**Symptoms, signs, etc. not elsewhere classified**			
Findings of drugs and substances, not normally in blood	Finding of alcohol in blood	10	R78.0
Findings of drugs and substances, not normally in blood	Finding of opiate in blood	10	R78.1
Findings of drugs and substances, not normally in blood	Finding of cocaine in blood	10	R78.2
Findings of drugs and substances, not normally in blood	Finding of hallucinogen in blood	10	R78.3
Findings of drugs and substances, not normally in blood	Finding of other drug addictive potential in blood	10	R78.4
Findings of drugs and substances, not normally in blood	Finding of psychotropic drug in blood	10	R78.5

**Injury, poisoning, other consequences of external causes**			
Poisoning by drugs, medicaments, biological substances	by narcotics and psychodysleptics	10	T40
Poisoning by drugs, medicaments, biological substances	by barbiturates	10	T42.3
Poisoning by drugs, medicaments, biological substances	by benzodiazepines	10	T42.4
Poisoning by drugs, medicaments, biological substances	by other antiepileptic and sedative-hypnotic drugs	10	T42.6
Poisoning by drugs, medicaments, biological substances	by unspecified sedative-hypnotic drugs	10	T42.7
Poisoning by drugs, medicaments, biological substances	Poisoning by psychotropic drugs, other	10	T43
Poisoning by drugs, medicaments, biological substances	by alcohol deterrent	10	T50.6
Poisoning by drugs, medicaments, biological substances	by opiate antagonist	10	T50.7
Poisoning by drugs, medicaments, biological substances	Toxic effect of alcohol	10	T51
Poisoning by drugs, medicaments, biological substances	Toxic effect of organic solvents	10	T52
Poisoning by drugs, medicaments, biological substances	Toxic effect of aromatic hydrocarbons, etc. [CFCs]	10	T53
Poisoning by drugs, medicaments, biological substances	Toxic effect of other gases, fumes and vapours	10	T59
**External causes of morbidity and mortality**			
Accidental poisoning by noxious substances	by antiepileptic, sedative-hypnotic,psychotropic etc	10	X41
Accidental poisoning by noxious substances	by and exposure to narcotics and psychodysleptics	10	X42
Accidental poisoning by noxious substances	by alcohol	10	X45
Accidental poisoning by noxious substances	by organic solvents,hydrocarbons and their vapours	10	X46
Accidental poisoning by noxious substances	by other gases and vapours	10	X47
Intentional self-harm	by antiepileptic, sedative-hypnotic,psychotropic etc	10	X61
Intentional self-harm	by and exposure to narcotics and psychodysleptics	10	X62
Intentional self-harm	by alcohol	10	X65
Intentional self-harm	by organic solvents,hydrocarbons and their vapours	10	X66
Intentional self-harm	by other gases and vapours	10	X67
Event of undetermined intent	by antiepileptic, sedative-hypnotic,psychotropic etc	10	Y11
Event of undetermined intent	by and exposure to narcotics and psychodysleptics	10	Y12
Event of undetermined intent	by alcohol	10	Y15
Event of undetermined intent	by organic solvents,hydrocarbons and their vapours	10	Y16
Event of undetermined intent	by other gases and vapours	10	Y17
Supplementary factors related to causes of morbidity	Evidence of alcohol involvement, BAC	10	Y90
Supplementary factors related to causes of morbidity	Evidence of alcohol involvement, level intoxication	10	Y91
**Factors influencing health status, health service contact**			
Persons encountering health services, specific care	Alcohol rehabilitation	10	Z50.2
Persons encountering health services, specific care	Drug rehabilitation	10	Z50.3
Persons encountering health services, other circumstances	Alcohol abuse counselling and surveillance	10	Z71.4
Persons encountering health services, other circumstances	Drug abuse counselling and surveillance	10	Z71.5
Problems related to lifestyle	Alcohol use	10	Z72.1
Problems related to lifestyle	Drug use	10	Z72.2
Persons with potential health hazards	Personal history of psychoactive substance abuse	10	Z86.4

INJURY BY EXTERNAL CAUSES

Chapter / Diagnosis level 1	Diagnosis level 2	ICD ed.	ICD codes
**External causes** (excl. poisoning & medical comp.)			
Accidents	Transport accidents	9	E800-E848
	Accidents in the home (all entries under 'place of occurrence' = home)		E849.0
	Accidents (falls, fire, natural factors, drowning, other)		E880-E929
Accidents	Transport accidents	10	V01-V99<br>Y85
	Accidents (falls, mechanical forces, fire, natural factors, drowning, other)		W00-X39<br>X50-X59<br>Y86
Undetermined intent	Injury undetermined if accidental or purposely inflicted (excl. poisoning)	9	E983-E989
Undetermined intent	Injury undetermined if accidental or purposely inflicted (excl. poisoning)	10	Y20-Y34<br>Y87.2
Legal intervention	Injury due to legal intervention (e.g. manhandling)	9	E970-E977
Legal intervention	Injury due to legal intervention (e.g. manhandling)	10	Y35<br>Y89
**Assault** (excl. poisoning)			
Assault	Injury purposely inflicted by other persons (excl. by poisoning)	9	E960-E961<br>E963-E969
Assault	Assault (excl. by poisoning)	10	X91-Y09<br>Y87.1

INFECTION RELATED HOSPITALISATION

Chapter / Diagnosis level 1	Diagnosis level 2	ICD ed.	ICD codes
**Infections (any)**			
Infectious and parasitic diseases	Any hospitalisation	9	001-139
Infectious and parasitic diseases	Any hospitalisation	10	A00-B99
Respiratory system	Acute respiratory infections	9	460-466
Respiratory system	Acute respiratory infections	10	J00-J06
Respiratory system	Pneumonia and influenza	9	480-488
Respiratory system	Pneumonia and influenza	10	J09-J18
Factors influencing health service contact	Contact with and exposure to communicable diseases	9	V01
Factors influencing health service contact	Contact with and exposure to communicable diseases	10	Z20
Factors influencing health service contact	Carrier of infectious disease	9	V02
Factors influencing health service contact	Carrier of infectious disease	10	Z22
Factors influencing health service contact	Asymptomatic HIV status	9	V08
Factors influencing health service contact	Asymptomatic HIV status	10	Z21
**Infections in pregnancy**			
Complications related to pregnancy	Infections of the genitourinary tract in pregnancy	9	646.6
Complications related to pregnancy	Infections of the genitourinary tract in pregnancy	10	O23
Complications related to pregnancy	Infective and parasitic conditions in the mother	9	647
Complications related to pregnancy	Infective and parasitic conditions in the mother	10	O98
Complications related to pregnancy	Suspected fetal damage from maternal viral disease	10	O35.3
Certain conditions originating in the perinatal period	Fetus/newborn affected by maternal infectious and parasitic diseases	9	760.2
Certain conditions originating in the perinatal period	Fetus/newborn affected by maternal infectious and parasitic diseases	10	P00.2
Certain conditions originating in the perinatal period	Infections specific to the perinatal period	9	771
Certain conditions originating in the perinatal period	Infections specific to the perinatal period	10	P35-P39
**Chronic and sexually transmitted infections**			
Tuberculosis	Tuberculosis	9	010-018
Tuberculosis	Tuberculosis	10	A15-A19
HIV	HIV	9	042-044
HIV	HIV	10	B20-B24
Infections with a predominantly sexual mode of transmission	Syphilis and other venereal diseases	9	090-099
Infections with a predominantly sexual mode of transmission	Infections with a predominantly sexual mode of transmission	10	A50-A64
Herpes simplex	Herpes simplex	9	054
Herpes simplex	Herpes simplex	10	B00
Viral hepatitis	Viral hepatitis	9	070
Viral hepatitis	Viral hepatitis	10	B15-B19
Complications related to pregnancy	Infective/parasitic conditions in mother – Syphilis	9	647.0
Complications related to pregnancy	Infective/parasitic conditions in mother – Gonorrhea	9	647.1
Complications related to pregnancy	Infective/parasitic conditions in mother – Other venereal	9	647.2
Complications related to pregnancy	Infective/parasitic conditions in mother – Tuberculosis	9	647.3
Complications related to pregnancy	Infective/parasitic conditions in mother – Tuberculosis	10	O98.0
Complications related to pregnancy	Infective/parasitic conditions in mother – Syphilis	10	O98.1
Complications related to pregnancy	Infective/parasitic conditions in mother – Gonorrhea	10	O98.2
Complications related to pregnancy	Infective/parasitic conditions in mother – Other sexually trans.	10	O98.3
Complications related to pregnancy	Infective/parasitic conditions in mother – Viral hepatitis	10	O98.4
Complications related to pregnancy	Infective/parasitic conditions in mother – HIV	10	O98.7
Factors influencing health service contact	Asymptomatic HIV status	9	V08
Factors influencing health service contact	Asymptomatic HIV status	10	Z21
Factors influencing health service contact	Contact with or exposure to venereal diseases	9	V01.6
Factors influencing health service contact	Contact with or exposure to infections with sexual mode transmission	10	Z20.2
Factors influencing health service contact	Contact with or exposure to viral hepatitis	10	Z20.5
Factors influencing health service contact	Contact with or exposure to HIV	10	Z20.6
Factors influencing health service contact	Carrier of gonorrhoea	9	V02.7
Factors influencing health service contact	Carrier of other venereal diseases	9	V02.8
Factors influencing health service contact	Carrier of infections predominantly sexual mode	10	Z22.4
Factors influencing health service contact	Carrier of viral hepatitis	9	V02.6
Factors influencing health service contact	Carrier of viral hepatitis	10	Z22.5

MATERNAL MENTAL ILLNESS

Chapter / Diagnosis level 1	Diagnosis level 2	ICD	ICD codes
**Mental Health Service Presentation**			
Mental Health Service Presentation	Any MHIS contact (excl. substance-use related)<br>****exclude codes for alcohol or substances with abuse potential****	9-10	Any<br>**exc. Drug/Alc**
**Hospitalisation**			
Mental and behavioural disorders	Any hospitalisation (excl. substance-use related)<br>****exclude codes for alcohol or substances with abuse potential****	9	290-319<br>**exc. Drug/Alc**
Mental and behavioural disorders	Any hospitalisation (excl. substance-use related)<br>****exclude codes for alcohol or substances with abuse potential****	10	F00-F99<br>**exc. Drug/Alc**
Factors influencing health service contact	Personal history of a mental disorder	9	V11
Factors influencing health service contact	Mental and behavioural problems	9	V40
Factors influencing health service contact	Screening for mental disorders	9	V79
Factors influencing health service contact	General psychiatric examination	10	Z00.4<br>Z04.6
Factors influencing health service contact	Observation for suspected mental disorder	10	Z03.2
Complications mainly relating to pregnancy	Mental disorders complicating pregnancy, etc.	9	648.4
Complications mainly relating to pregnancy	Mental disorders complicating pregnancy, etc.	10	O99.3
**Self-harm** (excl. poisoning)			
Self-inflicted injury	Suicide and self-inflicted injury (excl. by poisoning)	9	E953-E959
Self-inflicted injury	Suicide and self-inflicted injury (excl. by poisoning)	10	X70-X84<br>Y87.0

PREGNANCY COMPLICATIONS

Chapter / Diagnosis level 1	Diagnosis level 2	ICD ed.	ICD codes
**Abruptio placentae & other placental disorders**			
Complications mainly related to pregnancy	Abruptio placentae	9	641.2
Complications mainly related to pregnancy	Other placental conditions affecting management of the mother	9	656.7
Maternal care related fetus, amniotic cavity, possible delivery probs	Abruptio placentae	10	O45
Maternal care related fetus, amniotic cavity, possible delivery probs	Placental disorders	10	O43
**Placenta previa**			
Complications mainly related to pregnancy	Placenta previa	9	641.0-641.1
Maternal care related fetus, amniotic cavity, possible delivery probs	Placenta previa	10	O44
**Premature Rupture of Membranes**			
Other indications for care in pregnancy, labor, and delivery	Premature rupture of membranes	9	658.1
Maternal care related fetus, amniotic cavity, possible delivery probs	Premature rupture of membranes	10	O42

MULTIPLE GESTATION

Chapter	Diagnosis level 1	Diagnosis 2	ICD ed.	ICD codes
Complications of pregnancy, etc.	Normal delivery, etc.	Multiple gestation	9	651
Supplementary classification	Live-born infants according to type	Twin birth mates liveborn	9	V31
Supplementary classification	Live-born infants according to type	Other multiples	9	V32-V37
Pregnancy, childbirth, puerperium	Maternal care related to the fetus	Multiple gestation	10	O30
Pregnancy, childbirth, puerperium	Maternal care related to the fetus	Complications to multiple gestation	10	O31
Pregnancy, childbirth, puerperium	Delivery	Multiple delivery	10	O84

CHROMOSOMAL ABNORMALITIES

Chapter	Diagnosis level 1	ICD ed.	ICD codes
Complications of pregnancy, childbirth, puerperium	Chromosomal anomalies	9	655.1
Congenital anomalies	Chromosomal anomalies	9	758
Pregnancy, childbirth and the puerperium	Maternal care for (suspected) chromosomal abnorm. in fetus	10	O35.1
Congenital malform., deform. and chromosomal ab.	Chromosomal abnormalities	10	Q90-Q99

NON-SIGNIFICANT FACTORS
(Tested alone and in combination)

Chapter / Diagnosis level 1	Diagnosis level 2	ICD ed.	ICD codes
**Cancer**			
Neoplasms	Malignant neoplasms	9	140-208
Neoplasms	Malignant neoplasms	10	C00-C97
Neoplasms	Neoplasms of uncertain or unspecified nature	9	235-239
Neoplasms	Neoplasms of uncertain or unspecified nature	10	D37-D48
**Anaemia**			
Diseases of the blood and blood-forming organs	Anemia	9	280-285
Diseases of the blood and blood-forming organs	Anemia	10	D50-D64
Complications mainly related to pregnancy	Anemia complicating pregnancy	9	648.2
Complications mainly related to pregnancy	Anemia complicating pregnancy	10	O99.0
**Nutritional deficiencies**			
Endocrine, nutritional and metabolic diseases	Nutritional deficiencies	9	260-269
Endocrine, nutritional and metabolic diseases	Nutritional deficiencies	10	E50-E64
**Malnutrition**			
Endocrine, nutritional and metabolic diseases	Malnutrition	10	E40-E46
Maternal disorders related to pregnancy	Malnutrition in pregnancy	10	O25
Complications predominantly related to pregnancy	Maternal care for low weight gain in pregnancy	10	O26.1
**Obesity**			
Endocrine, nutritional and metabolic diseases	Obesity	9	278.0-278.1
Endocrine, nutritional and metabolic diseases	Obesity	10	E65-E66
Complications predominantly related to pregnancy	Maternal care for excessive weight gain in pregnancy	10	O26.0
**Diabetes mellitus**			
Endocrine, nutritional and metabolic diseases	Diabetes mellitus	9	250
Endocrine, nutritional and metabolic diseases	Diabetes mellitus	10	E10-E14
Complications mainly related to pregnancy	Diabetes mellitus in pregnancy	9	648.0
Complications mainly related to pregnancy	Diabetes mellitus in pregnancy	10	O24
Complications mainly related to pregnancy	Abnormal glucose tolerance complicating pregnancy	9	648.8
Certain conditions originating in the perinatal period	Syndrome of 'infant of a diabetic mother'	9	755.0
**Abortion (miscarriage and induced)**			
Complications of pregnancy	Ectopic and molar pregnancy	9	630-633
Complications of pregnancy	Other pregnancy with abortive outcome	9	634-639
Complications of pregnancy	Pregnancy with abortive outcome	10	O00-O08
Complications of pregnancy	Pregnancy care of habitual aborter	10	O26.2
**Hypertension**			
Complications mainly related to pregnancy	Hypertension complicating pregnancy, childbirth and the puerperium	9	642.0<br>642.2-642.3
Hypertensive disorders in pregnancy	Pre-existing hypertension complicating pregnancy, childbirth, puerperium	10	O10
Hypertensive disorders in pregnancy	Gestational hypertension	10	O13
Hypertensive disorders in pregnancy	Unspecified maternal hypertension	10	O16
**Preeclampsia**			
Complications mainly related to pregnancy	Preeclampsia	9	642.4-642.5
Complications mainly related to pregnancy	Preeclampsia or exclampsia superimposed on chronic hypertension	9	642.7
Hypertensive disorders in pregnancy	Preeclampsia superimposed on chronic hypertension	10	O11
Hypertensive disorders in pregnancy	Preeclampsia	10	O14
**Eclampsia**			
Complications mainly related to pregnancy	Eclampsia	9	642.6
Hypertensive disorders in pregnancy	Eclampsia	10	O15
**Renal disease**			
Genitourinary system	Renal failure	9	584-586
Genitourinary system	Renal failure	10	N17-N19
Factors influencing health service contact	Encounter for dialysis and dialysis catheter care	9	V56
Complications mainly related to pregnancy	Hypertension secondary to renal disease complicating pregnancy	9	642.1
Complications mainly related to pregnancy	Unspecified renal disease in pregnancy without mention of hypertension	9	646.2
Factors influencing health service contact	Care involving dialysis	10	Z49
Maternal care other conditions predominantly related to pregnancy	Pregnancy related renal disease	10	O26.83
