# Supplementary material for: Determinants of infant mortality for children of women prisoners: a longitudinal linked data study
Source: BMC Pregnancy Childbirth. 2018 Jun 1;18:202. doi: 10.1186/s12884-018-1840-z (PMC5984779; doi:10.1186/s12884-018-1840-z)
Supplement: Supplementary file 2 — Regression models of infant mortality by main group for Indigenous children. Results of the multivariate regression conducted for each main grouping of variables to eliminate non-significant variables from including the final multivariate analysis. (PDF 330 kb) [file 12884_2018_1840_MOESM2_ESM.pdf]

Group 1 Demographic factors

Regression model of infant mortality, Indigenous children

		RR	[95%	CI]	*p*-value
Birth year		0.97	0.95	0.98	<.001
Sex					
	Male	1.22	1.01	1.47	.040
	Female*				
Maternal Indigenous status					
	Indigenous	1.75	0.96	3.19	.066
	Non-Indigenous*				
Socioeconomic status					
	Very low (<5%)	2.06	1.31	3.25	.002
	Low (6-25%)	1.93	1.24	2.99	.003
	Medium (26-50%)	1.79	1.12	2.85	.015
	High (51-100%)*				
Geographical remoteness					
	Major cities and inner regional*				
	Outer regional	1.10	0.84	1.45	.496
	Remote	0.96	0.73	1.27	.793
	Very remote	1.07	0.84	1.37	.578

* Reference category

(grey shading) Variables excluded from final multivariate model on basis of significance (*p*>.05)

\# Includes all other placental disorders except placenta previa.

Group 2 Baseline pregnancy risk factors

Regression model of infant mortality, Indigenous children

		RR	[95%	CI]	*p*-value
Multiple gestation					
	Yes	2.36	1.57	3.57	<.001
	No*				
Birth spacing					
	<18months	1.62	1.26	2.08	<.001
	Firstborn/18months+*				
Parity					
	Nulliparous	0.92	0.73	1.16	.486
	Parity 1-2				
	Parity 3+	1.22	0.97	1.53	.091

* Reference category

(shaded) Variables excluded from final multivariate model on basis of significance (p>.05)

Note. Maternal age was excluded prior to multivariate analysis as highly correlated with parity, which was more significant in univariate analysis

Group 3 Pregnancy complications

Regression model of infant mortality, Indigenous children

		RR	[95%	CI]	*p*-value
Abruptio placentae and other disorders[#]					
	Yes	3.73	1.89	7.34	<.001
	No*				
Placenta previa					
	Yes	2.10	0.69	6.42	.193
	No*				
Premature rupture of membranes					
	Yes	1.72	1.21	2.42	.002
	No*				
Infection related hospitalisation in pregnancy					
	Yes	0.99	0.70	1.40	.937
	No*				

* Reference category

(grey shading) Variables excluded from final multivariate model on basis of significance (p>.05)

[#] Includes all other placental disorders except placenta previa

Group 4 Other maternal factors/exposures in pregnancy

Regression model of infant mortality, Indigenous children

		RR	[95%	CI]	*p*-value
Substance use related service contact in pregnancy					
	Yes	1.99	1.17	3.36	.011
	No*				
Hospitalisation for external causes of injury in pregnancy					
	Yes	1.43	1.02	1.99	.038
	No*				
Mental health related service contact in pregnancy					
	Yes	0.70	0.39	1.27	.243
	No*				
Sibling in contact with Child Protection in pregnancy					
	Yes	1.97	1.36	2.86	<.001
	No*				

* Reference category

(shaded) Variables excluded from final multivariate model on basis of significance (p>.05)
